# A Risk Stratification Model for Lung Cancer Based on Gene Coexpression Network and Deep Learning

**DOI:** 10.1155/2018/2914280

**Published:** 2018-01-16

**Authors:** Hongyoon Choi, Kwon Joong Na

**Affiliations:** ^1^Cheonan Public Health Center, Chungnam, Republic of Korea; ^2^Department of Community Health, Korea Health Promotion Institute, Seoul, Republic of Korea; ^3^Department of Clinical Medical Sciences, Seoul National University, College of Medicine, Seoul, Republic of Korea

## Abstract

Risk stratification model for lung cancer with gene expression profile is of great interest. Instead of previous models based on individual prognostic genes, we aimed to develop a novel system-level risk stratification model for lung adenocarcinoma based on gene coexpression network. Using multiple microarray, gene coexpression network analysis was performed to identify survival-related networks. A deep learning based risk stratification model was constructed with representative genes of these networks. The model was validated in two test sets. Survival analysis was performed using the output of the model to evaluate whether it could predict patients' survival independent of clinicopathological variables. Five networks were significantly associated with patients' survival. Considering prognostic significance and representativeness, genes of the two survival-related networks were selected for input of the model. The output of the model was significantly associated with patients' survival in two test sets and training set (*p* < 0.00001, *p* < 0.0001 and *p* = 0.02 for training and test sets 1 and 2, resp.). In multivariate analyses, the model was associated with patients' prognosis independent of other clinicopathological features. Our study presents a new perspective on incorporating gene coexpression networks into the gene expression signature and clinical application of deep learning in genomic data science for prognosis prediction.

## 1. Introduction

Risk stratification based on gene expression profiles is of major biomedical interest in lung cancer research [1–6]. Previous studies developed risk stratification models that mostly focused on individual prognostic genes. However, these studies have not fully considered the nature of biological networks and their systematic properties. Since it is more evident that biological processes are derived from numerous interactions between many cellular components, gene network analysis could provide valuable information of cancer pathogenesis [7]. Among the various biological networks, gene coexpression network has some strengths: not relying on prior information about genes, avoiding biologically wrong assumptions about independence of gene expression levels, and alleviating multiple testing problems [8].

Lung cancer, mainly, non-small-cell lung cancer, is one of the most common cancers and is the leading cause of cancer-related death worldwide [9, 10]. Currently, TNM staging system is a universal guideline for prognosis prediction and treatment decision. However, heterogeneous molecular features of lung cancer require diverse adjuvant treatment options and lead to different prognosis even in the same stage [11]. Hence, there has been a constant need for developing better risk stratification models to predict accurate prognosis and to improve cancer-related survival.

The main objectives of this study were (1) to identify survival-related gene coexpression network modules (2) and to propose a deep learning- (DL-) based risk stratification model reflecting survival-related network modules. Using public microarray datasets from the Gene Expression Omnibus (GEO), we identified survival-related network modules of lung adenocarcinoma. Subsequently, we constructed DL-based prognostic score using representative genes of survival-related network modules and it showed great prognostic property in all cohorts.

## 2. Materials and Methods

### 2.1. Gene Expression Data

Total eleven microarray datasets from NCBI GEO were included in the study. Two datasets with survival information were set as independent test sets and the others were merged and set as training set (Supplementary Table 1). Detailed preprocessing methods are described in Supplementary Methods (available here).

### 2.2. Weighted Gene Coexpression Network Construction from the Training Set

**Figure 1:**
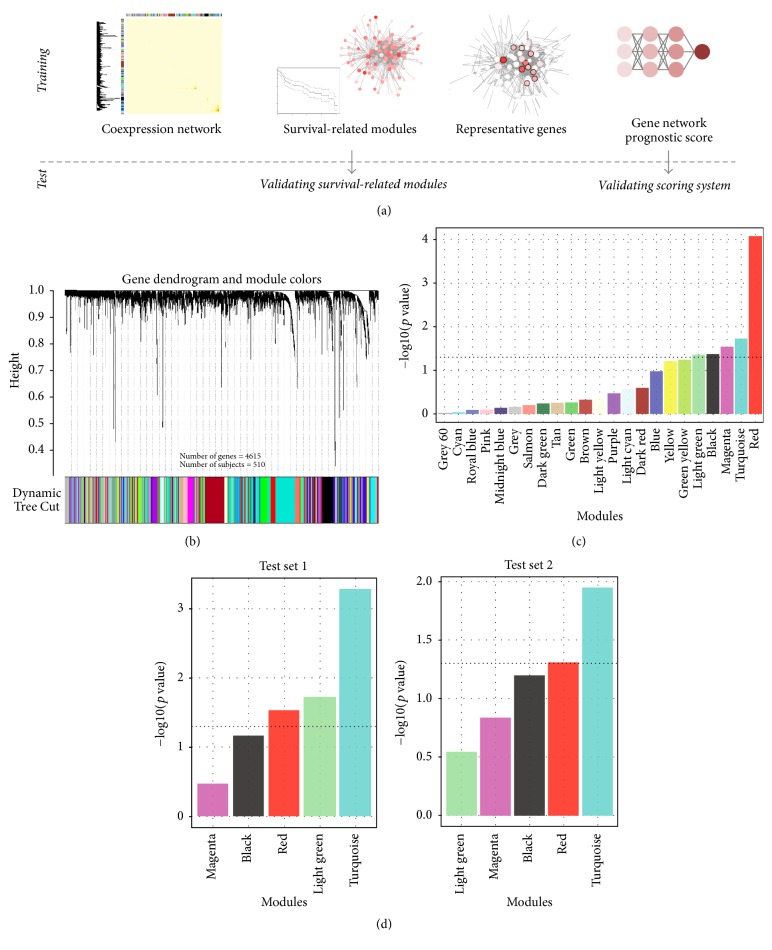
*Gene coexpression network construction and survival-related modules identification*. (a) A schematic diagram summarizing our risk stratification modeling strategy. Gene coexpression network was constructed from the training set. Gene network modules were extracted based on topological overlap. Survival-related modules were identified from the training set and validated in the two test sets. We selected representative genes from survival-related modules, and built network-based prognostic scoring system using deep learning. (b) Gene dendrogram and modules identified by weighted gene coexpression network analysis from the training set. Modules were labeled with different colors. (c) Univariate Cox regression analysis of module eigengene in the training set was performed. Module eigengene is a representative expression value of genes of each module calculated by the principal component analysis. The dotted line represents cutoff value (*p* value = 0.05) for significance, and five modules were identified as survival-related network modules. (d) Survival-related network modules were validated in the two test sets using Cox regression analysis. Three modules from test set 1 and two modules from test set 2 were significantly associated with overall survival.

We used weighted gene coexpression network analysis (WGCNA) package [8, 12] to build a weighted gene coexpression network from the training set. We created a correlation matrix on the basis of Pearson's correlation coefficient for all pairwise genes across all samples. The power, the key parameter for weighted network, was selected to optimize both the scale-free topology and sufficient node connectivity and we chose a threshold of 6 in this study (Supplementary Figure 1). The correlation matrix was transformed into adjacency matrix using the power function, and pairwise topological overlap (TO) between genes was calculated. We identified network modules using hierarchical clustering method with TO dissimilarity as the distance measure. The modules were detected using dynamic tree cut algorithm [13], defining height cutoff value of 0.99, deep split as 2, and minimum module size cutoff value of 30. Genes that were not assigned to any module were classified to color gray (Figure 1).

### 2.3. Identification and Validation of Survival-Related Network Modules

For each module, we summarized the module expression profile by module eigengene (ME), which is the first principal component of the expression matrix of the corresponding module. We used ME as the representative of each module to evaluate association with overall survival (OS). The survival-related network modules were identified using Cox regression analysis in the training set. For validation, the same genes included in the network construction were extracted from each test set. ME was calculated based on the expression profile of each test set, and the association between ME and OS was evaluated using Cox regression analysis to see whether the modules identified from the training set are also associated with OS in each test set. The modules with uncorrected *p* value under 0.05 were regarded as significant survival-related network modules. We functionally annotated all survival-related network modules with gene ontology biological process terms using hypergeometric test (Supplementary Methods).

### 2.4. DL-Based Risk Stratification Model

To simplify risk stratification model, we selected representative genes of the survival-related modules for model construction. Representativeness of a gene was measured by gene module membership (GMM), a correlation coefficient between gene expression profile and ME of given module. Expression profiles of representative genes were used for the input of the DL because they were expected to preserve coexpression patterns and to reflect the systematic properties of survival-related network modules. Convolutional neural network (CNN) was specifically used to extract gene expression patterns of modules. It finally produced gene network prognostic score (NetScore). Details of selection of representative genes and architecture of DL framework are described in Supplementary Methods.

The DL-based risk stratification model was generated using patients' data of the training set. Parameters related to training of the neural network including number of layers, nodes, training epoch and learning rate were determined by 5-fold cross-validation. Training set was randomly divided into 5 subsets. At each step, a single subset was left for testing and other four subsets were used for training. The performance of the model was measured by Harrell's* C*-index of the final output score of the model [14]. The optimal parameters were selected according to the maximum average* C*-index across the 5-fold of the loop. The predictive value of NetScore was independently validated in two test sets.* C*-index for each test set was also evaluated.

### 2.5. Survival Analysis Using NetScore in All Cohorts

Prognostic property of NetScore as a continuous variable was evaluated by univariate Cox analysis. To define risk groups, NetScore was dichotomized using the median value in each cohort. Kaplan-Meier method was used to assess survival rates according to the risk groups and survival rate differences were assessed with the log-rank test. Additionally, independent prognostic value of NetScore was assessed by multivariate and subgroup analysis. Multivariate Cox analysis was performed using clinical and pathological variables as well as NetScore. Subgroups were divided on the basis of clinical and pathological features, and univariate Cox analysis of NetScore was performed in each subgroup.

## 3. Results

### 3.1. Gene Coexpression Network Modules from the Training Set

We aimed at developing a risk stratification model based on gene coexpression networks (Figure 1(a)). The networks were constructed from the training set which consists of microarray data of 510 lung adenocarcinoma samples. The clinicopathological features of all samples from the training set are detailed in Supplementary Table 2. Using WGCNA, 23 coexpression network modules were identified from the training set (Figure 1(b)). The relationship between modules is visualized with hierarchical clustering dendrogram and heatmap of the corresponding ME (Supplementary Figure 2).

### 3.2. Identification of Survival-Related Modules from the Training Set and Validation in Test Sets

Total five modules were significantly associated with OS (Figure 1(c)): red (*p* < 0.0001), turquoise (*p* = 0.018), magenta (*p* = 0.029), black (*p* = 0.043), and light green (*p* = 0.044). To validate the survival-related modules, we conducted survival analysis in two independent test sets (GSE31210 as test set 1 and GSE30219 as test set 2; *n* = 226 and 84, resp.). Consequently, turquoise (*p* = 0.0005), light green (*p* = 0.019), and red (*p* = 0.030) modules in test set 1 and turquoise (*p* = 0.011) and red (*p* = 0.049) modules in test set 2 were significantly associated with OS (Figure 1(d)).

**Figure 2:**
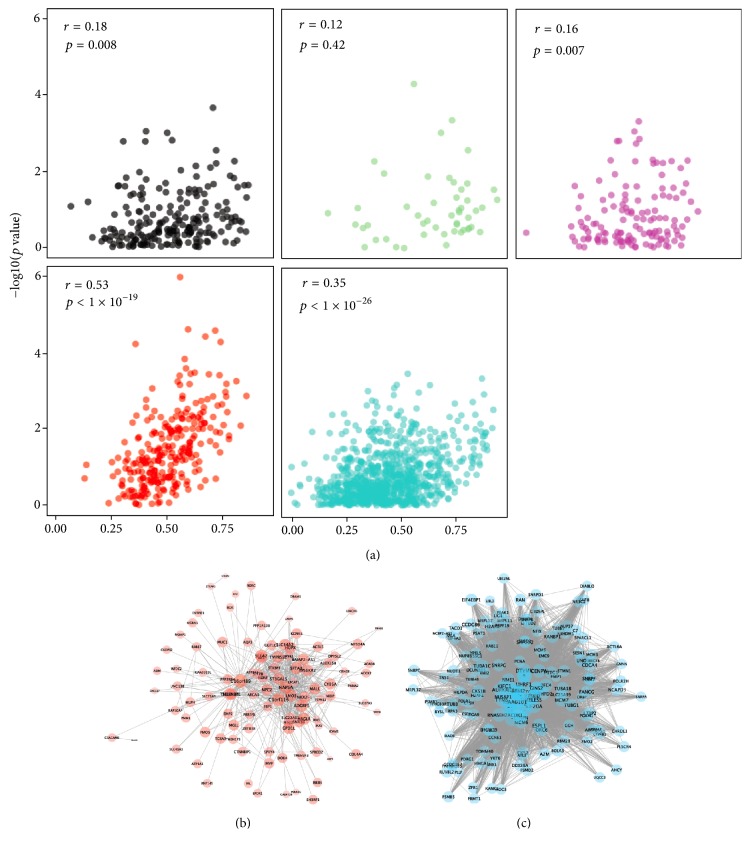
*Selection of representative genes of survival-related network modules*. (a) To construct risk stratification model, representative genes were selected according to the gene module membership. Gene module membership was correlated with the significance of association between individual gene expression and survival. *y*-axis represents statistical significance calculated by univariate Cox analysis of individual genes. A strong correlation was found in the red and turquoise modules (*r* = 0.53 and *p* < 1 × 10^−19^ for red module; *r* = 0.35 and *p* < 1 × 10^−23^ for turquoise module). Coexpression networks of red (b) and turquoise (c) modules were visualized. Note that 160 genes among 880 genes of turquoise module and their connections were shown. 160 genes were selected according to the gene module membership. Size of nodes is proportional to gene module membership.

The networks of two common survival-related network modules (red and turquoise) are presented in Figures 2(a) and 2(b). The significantly enriched gene ontology terms of the red module included “organic acid catabolic process,” “carboxylic acid catabolic process,” and “small molecule catabolic process,” and the turquoise module included “DNA strand elongation involved in DNA replication,” “mitotic cell cycle phase transition,” “DNA-dependent DNA replication” (Supplementary Table 3).

### 3.3. DL-Based Risk Stratification Model Using Representative Genes of Survival-Related Module

By measuring the correlation between gene significance for OS (*p* value) and GMM in each survival-related module, we identified two modules demonstrating high correlation with statistical significance (*r* = 0.53, *p* < 1 × 10^−19^ and *r* = 0.35, *p* < 1 × 10^−26^ for red and turquoise module, resp.; Figure 2(c)). Based on the strong correlation, we could assume that the genes with high representativeness measured by GMM have high significance for OS and are the most important elements of the module; therefore, we selected top 10 genes according to GMM from the red and turquoise modules for the DL-based risk stratification model construction (Supplementary Figure 3).

**Figure 3:**
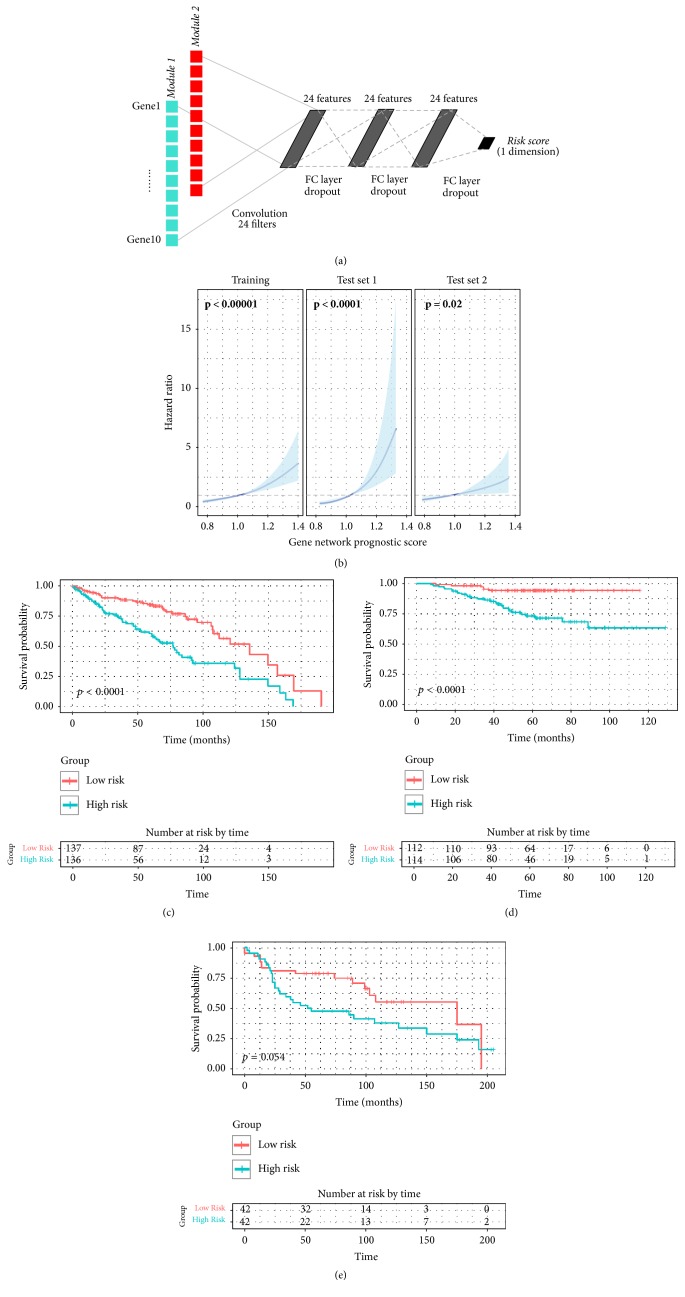
*Risk stratification model using representative genes of survival-related network modules*. (a) To construct risk stratification model, deep convolutional neural network was used. Input data were expression value of top 10 genes from each of red and turquoise module. The first layer consists of one-dimensional convolutional filters which extract gene expression patterns of each module. Three additional fully connected (FC) layers were followed and connected to the output score gene network prognostic score (NetScore). Detailed training process and architecture of the neural network are described in Supplementary Methods. (b) Univariate Cox regression analysis of NetScore as a continuous variable was performed in the training and two test sets. It shows significant association between the score and overall survival in all sets. The blue line represents hazard ratio for overall survival and the blue area represents 95% confidence interval. (c–e) Overall survival of dichotomized group according to NetScore was depicted by Kaplan-Meier survival curve. The statistical difference was tested by log-rank test. The high-risk group showed worse survival in the training set (c) and test set 1 (d) with statistical significance. The high-risk group of the test set 2 (e) also showed worse prognosis though the difference did not reach statistical significance.

The expression profiles of selected 20 genes were used as input data of the risk stratification model (Figure 3(a)). NetScore, the final output of our model, was significantly associated with OS in the training and two test sets (Figure 3(b)) (*p* < 0.00001, *p* < 0.0001 and *p* = 0.02 for training set and test sets 1 and 2, resp.). Subjects were divided into two groups, high- and low-risk groups, according to the median value of NetScore in each cohort. The high-risk group was significantly associated with OS in the training set (*p* < 0.0001; Figure 3(c)) and in test set 1 (*p* < 0.0001; Figure 3(d)). A trend of the association was also shown in test set 2 (*p* = 0.054; Figure 3(e)).

### 3.4. NetScore as an Independent Predictive Factor for Prognosis

**Figure 4:**
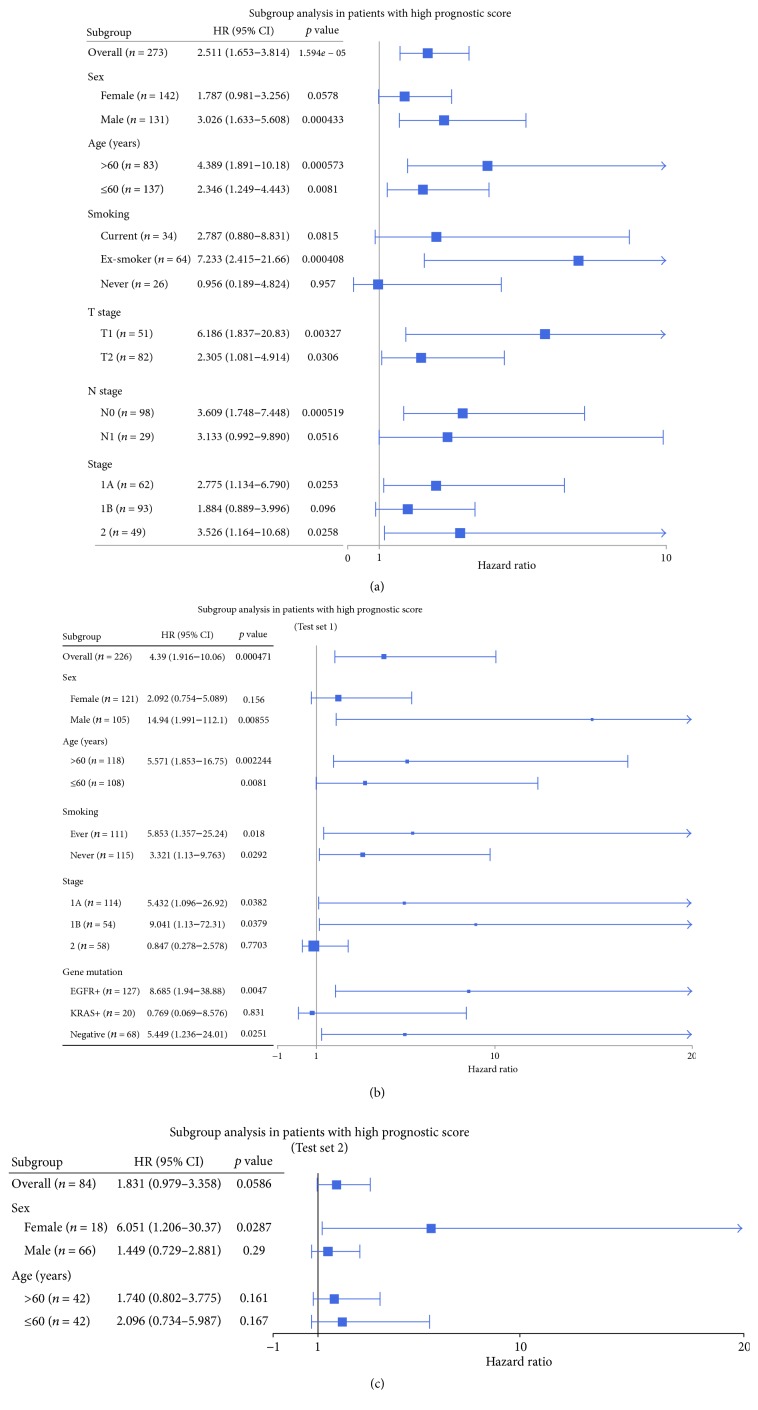
*Subgroup analysis using NetScore*. (a) Predictive value of our risk stratification model was tested in subgroups classified by clinicopathological characteristics of the training set. A trend of association between the risk group and overall survival was found in all subgroups. (b, c) The same subgroup survival analysis was also performed in both test sets. (b) The risk group was associated with overall survival regardless of clinicopathological variables except female, stage II, and KRAS mutation subgroups in test set 1. (c) Regardless of subgroups, a trend of poor prognosis in high-risk group was also found in test set 2.

Cox multivariate analysis revealed that the risk group was associated with OS independent of stage as well as other clinicopathological features in the training set and test set 1 (Table 1). The independent predictive factors for OS in Cox multivariate analysis were the risk group (*p* = 0.001) and T-stage 3 (*p* = 0.030) in training set and the risk group (*p* = 0.01) and EGFR mutation status (*p* = 0.005) in test set 1. In test set 2, there was no feature significantly associated with OS in univariate Cox analysis, though the high-risk group showed a trend of unfavorable prognosis (*p* = 0.06). We also evaluated the prognostic value of NetScore in subgroups divided by clinical and pathological features. In the training set, the high-risk group was significantly associated with poor prognosis in subgroups regardless of age and T-stage. In all subgroups, a trend of close relationship between the risk group and OS was found except never-smoking subgroup (Figure 4(a), Supplementary Figure 4). According to subgroup analysis in test set 1, the risk group was closely associated with OS in male, old-aged, ever/never smokers, stage IA/IB, EGFR positive and all negative mutation subgroups (Figure 4(b), Supplementary Figure 5). A trend of association between the risk group and OS was also revealed in each subgroup of test set 2, regardless of clinical features including sex, age, and T-stage (Figure 4(c), Supplementary Figure 6).

## 4. Discussion

In this study, we developed a risk stratification model for lung adenocarcinoma based on gene coexpression networks and deep learning. Survival-related network modules were identified in multiple cohorts and representative genes of these modules were selected for risk stratification modeling. The model constructed by deep CNN reflects gene expression patterns of survival-related network modules and it provides prognostic score, NetScore. The NetScore was significantly associated with OS in all cohorts and also an independent predictor for OS from clinicopathological variables.

The model based on survival-related network modules can provide more robust risk stratification compared with models focusing on statistical combination of individual prognostic genes which have been proposed in the previous studies [1–6]. In spite of previous promising results of individual gene-based models, they failed to validate in independent samples of other study [4]. Furthermore, there were few overlapping significant prognostic genes in the previous models. A meta-analysis of published gene expression data revealed that few genes were associated with survival of lung adenocarcinoma [15]. The result of few significant prognostic genes in large samples implied the limitation of usage of individual genes for risk stratification. Besides, selection of individual significant genes has a substantial problem of multiple statistical testing [16]. Instead of these previous approaches, systemic approach integrating gene interaction as well as individual genes would be a breakthrough for robust risk stratification modeling because variation patterns of their expression levels can be associated with prognosis.

Recently, DL has dramatically improved data analysis in genomics and imaging fields [17, 18]. The main contribution of DL for our risk stratification model is to apply deep neural network to gene expression data. It employed convolutional layers for extracting multiple gene expression patterns. Another contribution is to solve regression problems of survival data by using a specialized loss function [19] (see Supplementary Methods). We compared predictive accuracy of DL-based model and conventional Cox proportional hazard model obtained from the expression level of selected 20 genes. Predictability of the DL-based model was significantly higher than that of the Cox model in test set 1 (*C*-index = 0.709 ± 0.042 and 0.608 ± 0.046, resp.; *p* = 0.004). It was also higher in the training set and test set 2 though the difference did not reach statistical significance (Supplementary Methods, Supplementary Figure 7). Furthermore, to confirm robustness of NetScore, the model was retrained by the dataset combined by original training and test set 1 and validated in test set 2. NetScore of the retrained model was also significantly associated with OS in the test set 2 (*p* = 0.003;* C*-index = 0.651 ± 0.042). To our knowledge, NetScore is the first study that apply deep convolutional neural network to high-dimensional gene expression data for predicting prognosis. By applying this novel approach to various genomic data, risk stratification and survival prediction could be improved compared with conventional Cox model.

NetScore was trained by various samples with different clinicopathological characteristics. We found NetScore was associated with sex, smoking status, stage, and molecular subtypes (Supplementary Figure 8). Briefly, a trend of high NetScore was found in male, smokers, late stage, and KRAS mutation positive samples. Nonetheless, NetScore was significantly associated with OS independent of clinicopathological variables according to multivariate and subgroup analyses. Of note, NetScore was significant predictor in early stage subgroups (stage IA/IB). This finding could be important because the new risk stratification could identify patients who might need adjuvant chemotherapy. For example, a recent clinical trial using 15-gene signature based on individual prognostic genes showed successful selection of patients with stage IB and II NSCLC who would most likely benefit from adjuvant chemotherapy [20]. In the future, as a new prognostic biomarker based on gene network, the usefulness of NetScore should be tested whether it could affect clinical decision and compared with the previous prognostic models using individual genes.

We developed a risk stratification model for lung adenocarcinoma using gene coexpression network. A future extension of our work would be to apply this approach to the coexpression networks of other cancer types. In terms of technical improvement, modification of DL architecture and selection process of representative genes could improve the prediction accuracy. Finally, we expected that a prospectively designed clinical trial with well-controlled clinicopathological variables would help find clinical application of our new risk stratification model.

## Figures and Tables

**Table 1:** Univariate and multivariate Cox regression analysis of the risk stratification model and clinicopathological variables.

Variables	Univariate analysis		Multivariate analysis	
	Hazard ratio (95% CI)	*p* value	Hazard ratio (95% CI)	*p* value
*Training set*				
Gene network prognostic score, high risk group	2.51 (1.65–3.81)	<0.001	3.057 (1.556–6.006)	0.001
Age, older than 60	1.66 (0.96–2.86)	0.068		
Sex, male	1.28 (0.86–1.88)	0.221		
Smoking status, ex-smoker	0.59 (0.31–1.12)	0.108		
Smoking status, never smoker	0.51 (0.22–1.19)	0.120		
T stage: II	2.50 (1.31–4.79)	0.006	1.266 (0.599–2.674)	0.537
T stage: III	13.32 (2.89–61.32)	0.001	5.895 (1.189–29.237)	0.030
N stage: I	2.27 (1.28–4.05)	0.005	1.762 (0.943–3.294)	0.076

*Test set 1*				
Gene network prognostic score, high risk group	4.39 (1.92–10.06)	0.0004	2.97 (1.25–7.09)	0.01
Age, older than 60	1.27 (0.65–2.48)	0.49		
Sex, male	1.52 (0.78–2.96)	0.22		
Smoking status, never smoker	0.61 (0.31–1.19)	0.15		
Stage: II	4.23 (2.17–8.24)	0.00002		
EGFR mutation +	0.47 (0.24–0.93)	0.03	2.74 (1.36–5.54)	0.005
KRAS mutation +	0.87 (0.27–2.85)	0.82	0.64 (0.32–1.27)	0.20

*Test set 2*				
Gene network prognostic score, high risk group	1.81 (0.98–3.36)	0.06		
Age, older than 60	1.33 (0.73–2.43)	0.35		
Sex, male	0.83 (0.40–1.75)	0.63		
Stage: T2	1.65 (0.84–3.25)	0.14		

## Supplementary Materials

Supplementary Materials Supplementary Methods for public data collection and preprocessing, functional annotation and network visualization of survival-related network modules, representative genes selection for risk stratification model construction, comparison of predictability between DL-based model and conventional Cox proportional hazard model, and convolutional neural network for risk stratification. Supplementary Figure 1: gene coexpression network topology analysis for various soft threshold powers. Supplementary Figure 2: the relationship between coexpression network modules. Supplementary Figure 3: representative genes selected from the red and turquoise modules. Supplementary Figure 4: subgroup analysis using NetScore in the training set. Supplementary Figure 5: subgroup analysis using NetScore in the test set 1. Supplementary Figure 6: subgroup analysis using NetScore in the test set 2. Supplementary Figure 7: comparison of predictability between deep neural network and conventional Cox regression models. Supplementary Figure 8: association of gene network prognostic score (NetScore) with clinicopathological variables. Supplementary Table 1: microarray data sets from Gene Expression Omnibus used in this study. Supplementary Table 2: demographic and baseline clinical characteristics of patients. Supplementary Table 3: significantly enriched gene ontology biological process terms of five survival-related network modules.
