# Supplementary material for: A Risk Stratification Model for Lung Cancer Based on Gene Coexpression Network and Deep Learning
Source: Biomed Res Int. 2018 Jan 16;2018:2914280. doi: 10.1155/2018/2914280 (PMC5822793; doi:10.1155/2018/2914280)
Supplement: Supplementary Materials — Supplementary Methods for public data collection and preprocessing, functional annotation and network visualization of survival-related network modules, representative genes selection for risk stratification model construction, comparison of predictability between DL-based model and conventional Cox proportional hazard model, and convolutional neural network for risk stratification. Supplementary Figure 1: gene coexpression network topology analysis for various soft threshold powers. Supplementary Figure 2: the relationship between coexpression network modules. Supplementary Figure 3: representative genes selected from the red and turquoise modules. Supplementary Figure 4: subgroup analysis using NetScore in the training set. Supplementary Figure 5: subgroup analysis using NetScore in the test set 1. Supplementary Figure 6: subgroup analysis using NetScore in the test set 2. Supplementary Figure 7: comparison of predictability between deep neural network and conventional Cox regression models. Supplementary Figure 8: association of gene network prognostic score (NetScore) with clinicopathological variables. Supplementary Table 1: microarray data sets from Gene Expression Omnibus used in this study. Supplementary Table 2: demographic and baseline clinical characteristics of patients. Supplementary Table 3: significantly enriched gene ontology biological process terms of five survival-related network modules. [file 2914280.f1.pdf]

Supplementary Materials

A risk stratification model for lung cancer based on gene co-expression network and deep learning

[Contents]

Supplementary Methods

Supplementary Figures

Supplementary Tables

Supplementary References

Supplementary Methods

Public data collection and preprocessing

Microarray data sets were searched from the National Center for Biotechnology Information Gene Expression Omnibus (GEO) database (https://www.ncbi.nlm.nih.gov/geo/) [1] using keywords 'lung cancer', 'lung adenocarcinoma', or 'adenocarcinoma'. We searched for studies analyzed by single platform (Affymetrix HG-U133A Plus 2.0) in order to obtain high proportion of overlapping genes. In total, eleven microarray data sets were included and raw gene expression data were downloaded from the GEO data repository for preprocessing step [2-10]. We selected two microarray data sets with survival information (accession number GSE31210 [9] and GSE30219 [10]) as independent test sets, and the others [2-8] with or without survival information as the training set. For those microarray data sets containing multiple histologic types of lung cancer, only the samples from adenocarcinoma were extracted. Detailed information of data source used in this study can be found in **Supplementary Table 1**. All available clinico-pathological variables (age, sex, smoking status, stage, and molecular subtypes) and survival information (survival status and duration) were compiled from each microarray data sets using 'GEOquery' package [11] (**Table 1)**.

We generated the training set by assembling nine microarray data sets through stepwise preprocessing method described below. The raw gene expression data from microarray data sets were called and normalized using robust multichip average method using the 'affy'[12] package. On a study-by-study basis, we removed invalid and duplicated probe sets by 'featureFilter' function in 'genefilter' package [13] and mapped array probe sets for the respective gene symbols. In addition, to remove poor quality probes, we filtered out probe sets with low expression level (signal intensity < $\log_2(100)$ in at least 5% of samples within at least one study) and low variability (interquartile range < 0.5). As we combined microarray

data from different studies, we performed additional normalization using Combat algorithm [14] in order to eliminate potential batch effects. Lastly, we detected the outliers by calculating the inter-array correlation based on Pearson's correlation coefficient for all samples, and removed them. As a result, the training set contained 4615 probe sets from 510 lung adenocarcinoma samples including 273 samples with available survival information.

The raw gene expression data of both test sets were called and normalized as the same method with the training set. One outlier sample was removed from the test set 2; consequently, the test set 1 and 2 included 226 and 84 lung adenocarcinoma samples respectively.

Functional annotation and network visualization of survival-related network modules

The enrichment of the gene ontology terms in each module were evaluated based on the hypergeometric test using 'clusterProfiler'[15] package. The gene ontology biological process terms at false discovery rate under < 0.05 in each survival-related module were regarded as significantly enriched terms. The network of two common survival-related network modules (red and turquoise) was visualized with Cytoscape Software 3.4.0 [16].

Representative genes selection for risk stratification model construction

Representative genes of the survival-related network modules were selected to construct risk stratification model. Degree of representativeness of genes in each module was calculated by gene module membership (GMM), a correlation coefficient between gene expression profile and module eigengene. Additionally, the relationship between GMM and prognostic significance (p-value) of an individual gene was tested. Prognostic significance of gene was measured by univariate Cox regression analysis for overall survival. Pearson

correlation analysis was performed between GMM and prognostic significance for every gene. We selected top 10 genes according to the GMM from the modules which showed significant correlation between GMM and prognostic significance. Accordingly, expression levels of the selected genes in the same network module were highly correlated to each other, and they could be also highly associated with prognosis because of strong correlation between GMM and prognostic significance. The expression levels of selected genes were used for risk stratification model based on deep learning (DL).

Comparison of predictability between DL-based model and conventional Cox proportional hazard model

Expression level of all selected genes was fitted into multivariate Cox regression model and the predictive value of the Cox model was evaluated by C-index as in DL-based model. C-index of Cox model was measured by 5-fold cross validation in the training set, and it was calculated in two test sets. C-index of Cox model was compared with that of DL-based model in each cohort [17].

Convolutional neural network for risk stratification

DL framework was based on a nonlinear proportional hazard model, which assumed hazard function (λ), a product of a time-dependent baseline hazard function (λ_0) and a risk function determined by covariates: $\lambda(x,t) = \lambda_0(t) \times e^{h(x)}$. Conventional Cox model for the risk stratification using multiple covariates ($x_1, x_2, \dots, x_n$) estimates the risk function $h(x)$ by a combination of linear functions.

$$h_\beta(x) = \beta_1 x_1 + \beta_2 x_2 + \cdots + \beta_n x_n$$

DL-based risk stratification modeling also adopts proportional hazard model, however, replaces linear risk function with the output of neural network [18]. We designed a simple convolutional neural network (CNN) to estimate risk function, $h_\theta(x)$. Firstly, 1-dimensional convolutional filters were applied. Filter size was same as the input length, 10. Thus, the number of the output of the first layer was same as the number of convolutional filters. Genes in different modules were inputted as different channels. We set the number of filters were 24. The outputs of convolutional layer were hierarchically connected to three fully-connected (FC) layers. Each FC layer had 24 nodes except final output layer. For FC layers, a dropout function was applied to reduce overfitting and learn more robust features. This function randomly drops the connections with predefined probability. We set the probability as 0.5. The final output of CNN, $h_\theta(x)$, was a single node.

The CNN model was trained by the RMSprop algorithm [19]. The model was optimized to minimize the loss function, negative log partial likelihood.

$$L(\theta) = -\sum_{i:E_i=1}\left(h_\theta(x) - log\sum_{j\in R(T_i)} e^{h_\theta(x)}\right)$$

E_i=1 represents that the event has occurred in individual i at event time T_i. $j \in R(T_i)$ represents that another patient j is still at risk of the event at time T_i.

5-fold cross validation was performed to determine parameters of DL model. A randomly selected subset was used as an internal validation set and converging loss value C-index was monitored. Our framework was trained by initial learning rate with $1\text{x}10^{-4}$ and took 500 epochs for the training. The CNN was implemented using a deep learning library, Keras (ver. 1.0.4) with the Theano (ver. 0.8.2) backend [20].

Supplementary Figures

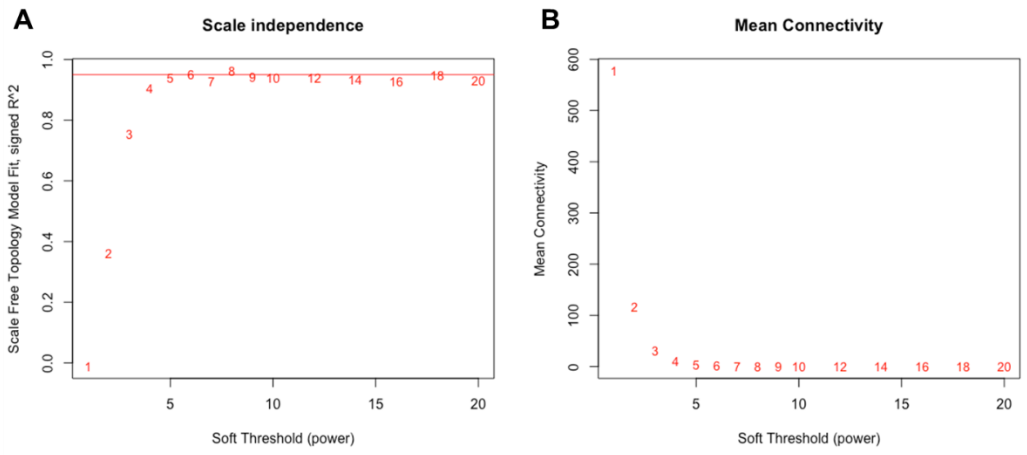

Supplementary Figure 1. Gene co-expression network topology analysis for various soft threshold powers. (A) Scale-free fit index was changed according to various powers of the correlation matrix of genes. The red line represents the cutoff value for the power ($R^2 = 0.95$) (B) We chose the smallest soft threshold power where the scale-free topology and mean connectivity seems to reach plateau.

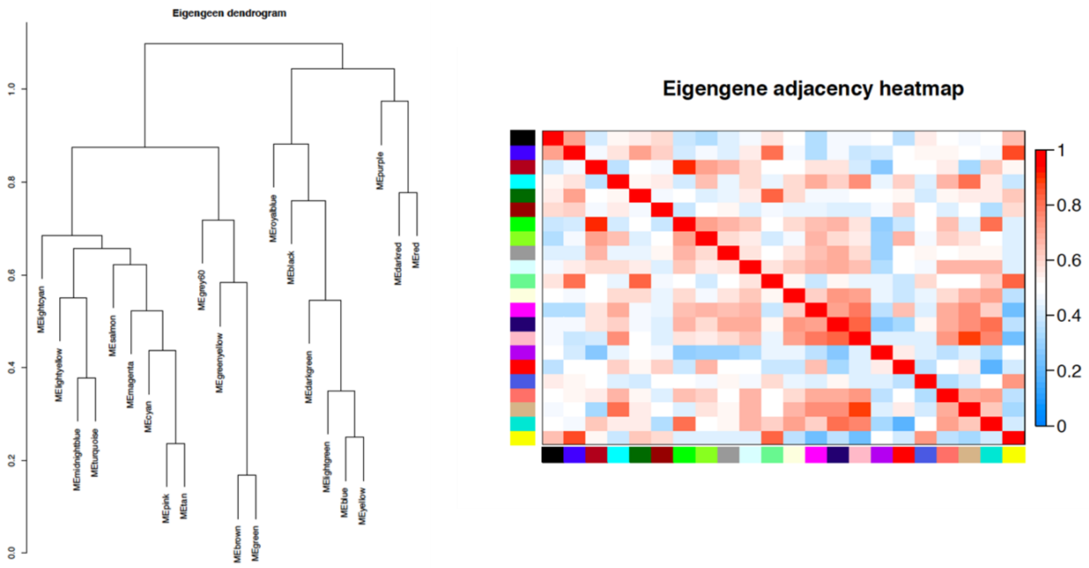

Supplementary Figure 2. The relationship between co-expression network modules. Module eigengene, the first principal component of each module, was calculated. The adjacency between modules was measured based on module eigengene and visualized by hierarchical clustering dendrogram (left panel) and heatmap (right panel).

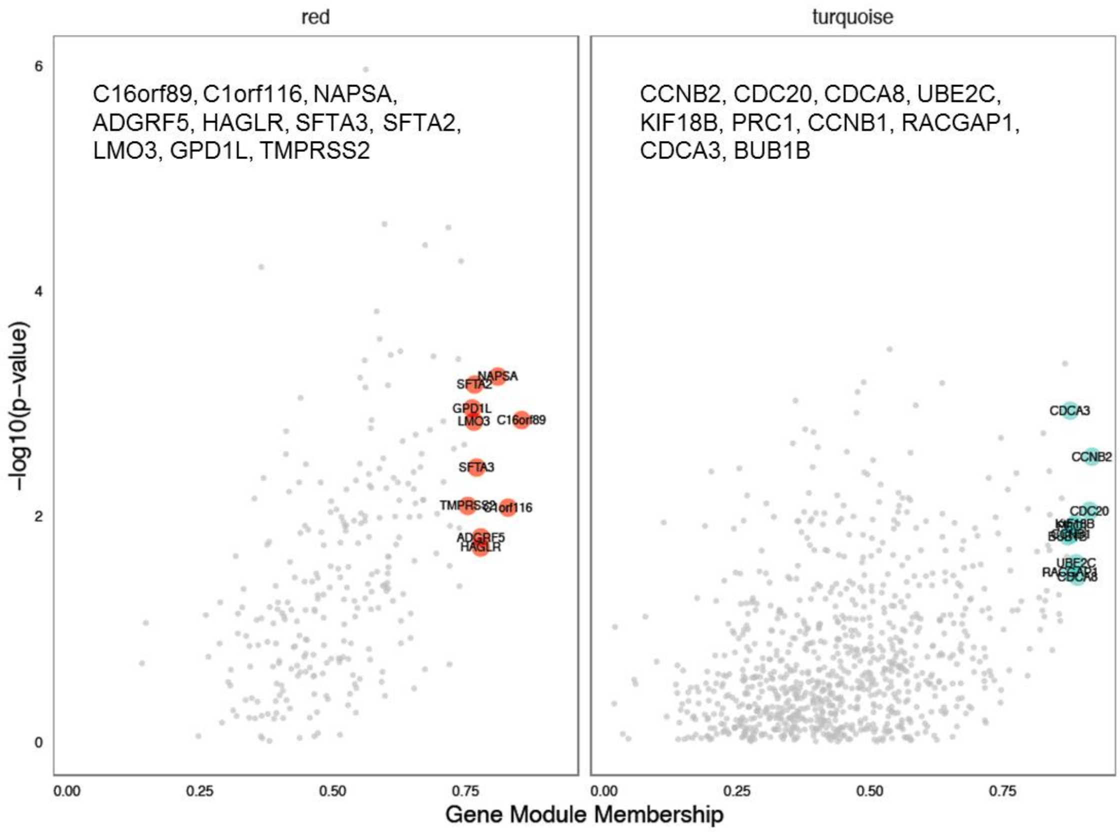

Supplementary Figure 3. Representative genes selected from the red and turquoise modules. Genes membered in each module was ordered according to the gene module membership (GMM). Genes in the red and turquoise modules showed strong correlation between GMM and the statistical significance (p-value) for the association with overall survival. Top 10 representative genes from each red and turquoise module according to GMM were selected for risk stratification model construction.

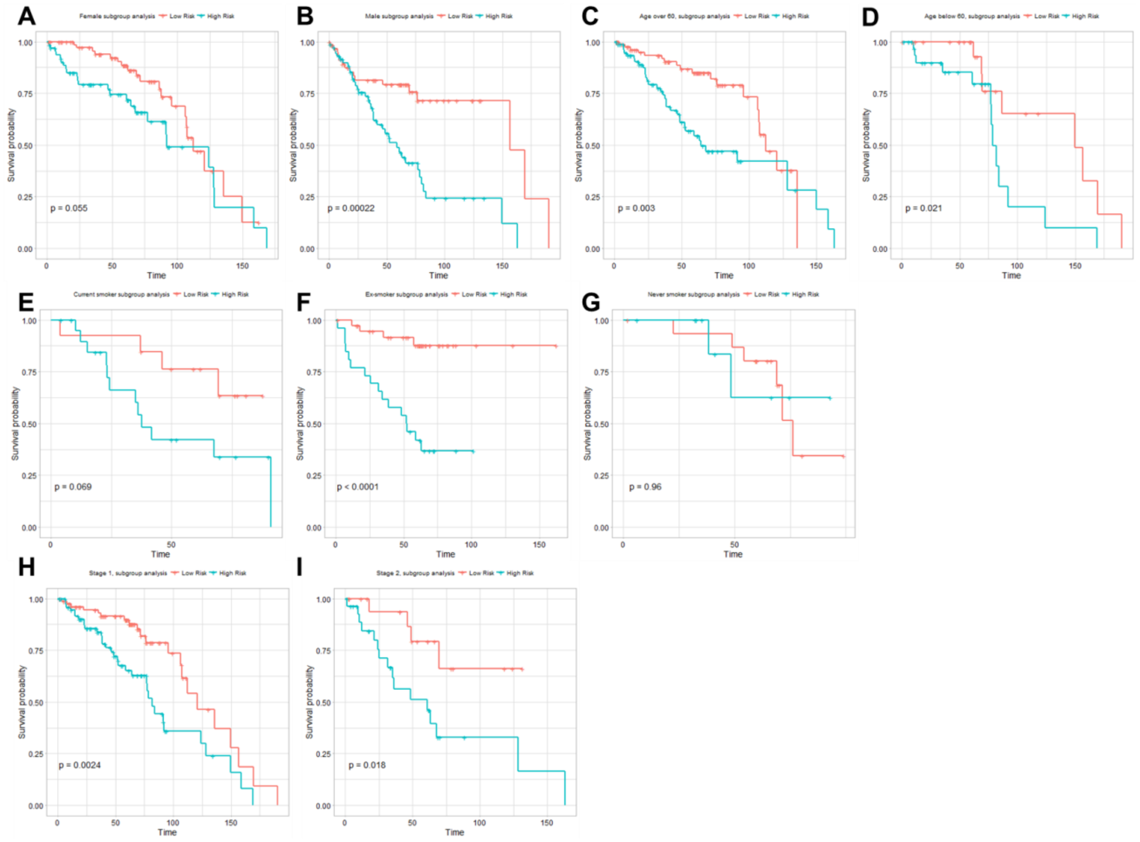

Supplementary Figure 4. Subgroup analysis using NetScore in the training set. Kaplan-Meier survival curve for overall survival according to the risk group dichotomized by the median value of NetScore: Female or male (A, B), old or young-aged group (older or younger than 60; C, D), current, ex- or never-smokers (E, F, G), and stage I or II (H, I). The statistical significance was tested by log-rank test.

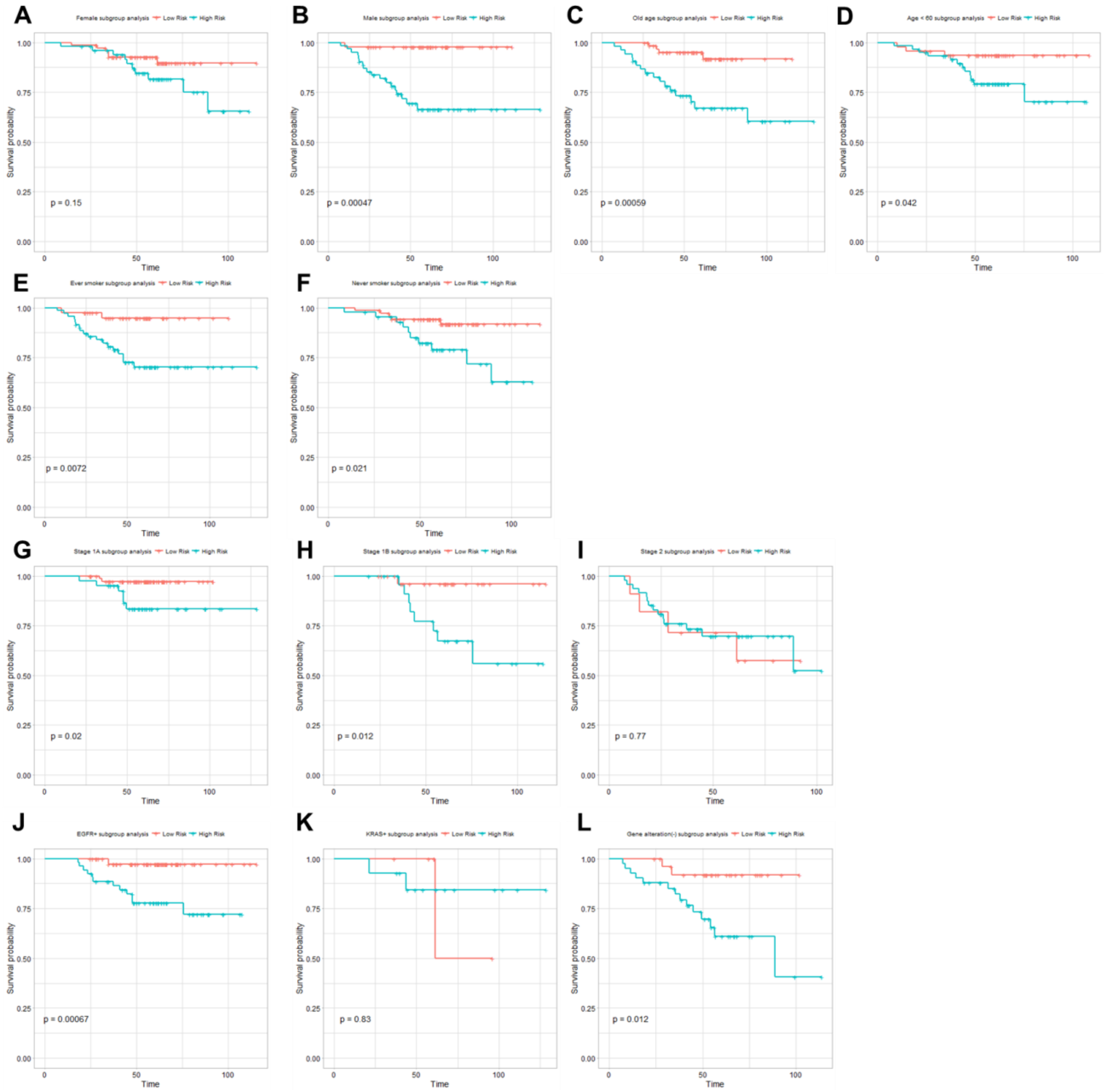

Supplementary Figure 5. Subgroup analysis using NetScore in the test set 1. Kaplan-Meier survival curve for overall survival according to the risk group dichotomized by the median value of NetScore: Female or male (A, B), old or young-aged group (older or younger than 60; C, D), ever- or never-smokers (E, F), stage IA, IB, or II (G, H, I), and EGFR mutation positive, KRAS mutation positive or all negative tumors (J, K, L). The statistical significance was tested by log-rank test.

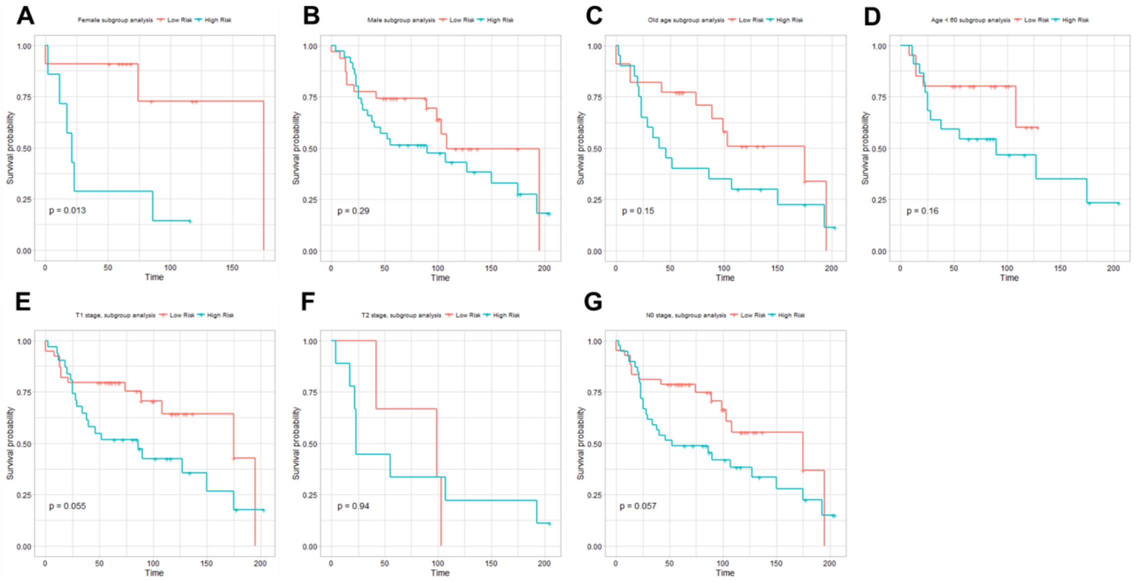

Supplementary Figure 6. Subgroup analysis using NetScore in the test set 2. Kaplan-Meier survival curve for overall survival according to the risk group dichotomized by the median value of NetScore: Female or male (A, B), old or young-aged group (older or younger than 60; C, D), T1 or T2 stage (E, F) and N0 stage (G). The statistical significance was tested by log-rank test.

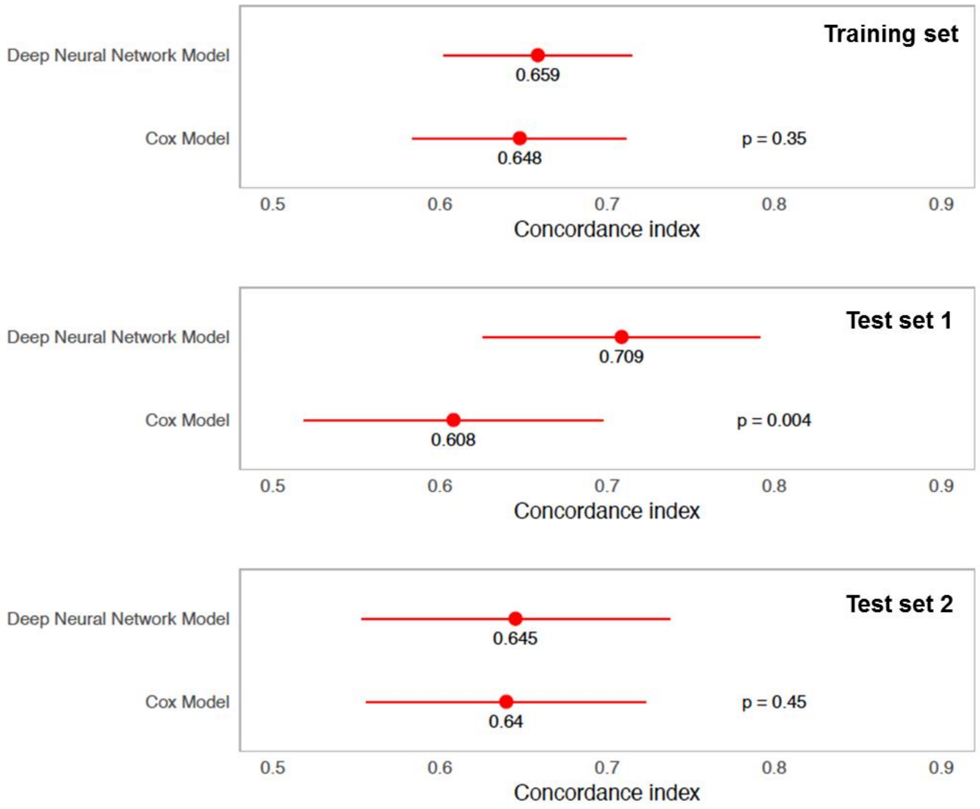

Supplementary Figure 7. Comparison of predictability between deep neural network and conventional Cox regression models. Both risk stratification models were generated using 20 representative genes. Our model was generated by a nonlinear proportional hazard model for the survival data based on deep convolutional neural network. The conventional model was generated by Cox proportional hazard model. Prediction accuracy of two models was statistically compared by concordance index (C-index). Our model based on deep neural network showed a trend of higher C-index than the conventional model in all cohorts. Red bars represent 95% confidence interval of C-indices.

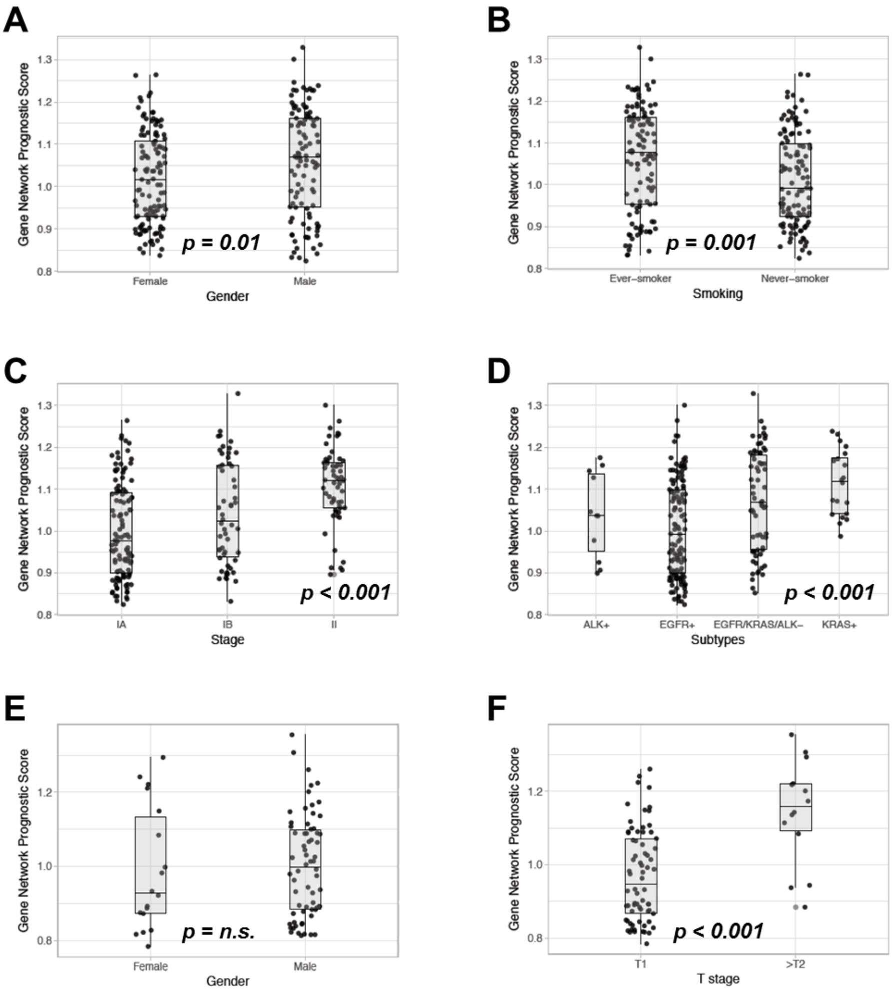

Supplementary Figure 8. Association of gene network prognostic score (NetScore) with clinico-pathological variables. The relationship between NetScore and clinico-pathological variables was tested in test set 1 (A-D) and 2 (E, F). (A) NetScore was significantly higher in male than female (1.06 ± 0.13 vs. 1.02 ± 0.11; t = 2.50; p = 0.01) and (B) ever-smoker than never-smoker (1.06 ± 0.12 vs. 1.01 ± 0.11; t=3.29; p = 0.001). (C) NetScore of stage IA, IB and II groups was significantly different (1.00 ± 0.11, 1.04 ± 0.12, and 1.11 ± 0.09, respectively; p < 0.001). (D) Subgroups according to the molecular subtypes showed significantly different NetScore (1.04 ± 0.10 for ALK fusion positive group, 1.01 ± 0.11 for EGFR mutation group, 1.11 ± 0.08 for KRAS mutation group and 1.07 ± 0.12 for all negative

group; $p < 0.001$). (E) In test set 2, NetScore of male and female patients was not significantly different (0.99 ± 0.17 for female and 1.00 ± 0.14 for male patients; $t= 0.26$; $p = 0.79$). (F) NetScore of pathological T2 or higher stage group was significantly higher than that of T1 stage group (1.14 ± 0.14 for T2 and 0.97 ± 0.13 for T1; $t= 4.26$; $p < 0.001$).

Supplementary Tables

Supplementary Table 1. Microarray data sets from Gene Expression Omnibus used in this study

Purpose	Accession number	Total sample size	Adenocarcinoma samples	Survival data availability	Survival data in adenocarcinoma
Training	GSE50081	181	127	yes	127
Training	GSE19188	156	45	yes	40
Training	GSE31546	17	17	yes	16
Training	GSE37745	196	106	yes	106
Training	GSE10245	58	40	no	
Training	GSE33532	100	40	no	
Training	GSE28571	100	50	no	
Training	GSE27716	40	40	no	
Training	GSE12667	75	68	no	
Test set 1	GSE31210	246	226	yes	226
Test set 2	GSE30219	307	85	yes	85

Supplementary Table 2. Demographic and baseline clinical characteristics of patients

Variables		Training set (n = 533)	*Available data*	**Test set 1** (GSE31210, n = 226)	**Test set 2** (GSE30219, n = 85)
**Sex**	Female : Male	229:209 (52.3%:47.7%)	438	121:105 (53.5%:46.5%)	19:66 (22.4%:77.6%)
**Age**		65.46 ± 10.14	313	59.58 ± 7.40	61.49 ± 9.28
**Smoking**	Current	52 (29.1%)	179	111(49.1%)	
	Ex-smoker	92 (51.4%)			
	Never	35 (19.5%)		115 (50.9%)	
**T stage**	T1	79 (43.2%)	183		71 (83.5%)
	T2	99 (54.1%)			12 (14.1%)
	T3	4 (2.2%)			2 (2.4%)
	T4	1 (0.5%)			
**N stage**	N0	136 (78.2%)	174		82 (96.5%)
	N1	34 (19.5%)			3 (3.5%)
	N2	4 (2.3%)			
**M stage**	M0	127 (100%)	127		85 (100%)
**Stage**	IA	91 (29.1%)	313	114 (50.4%)	
	IB	127 (40.6%)		54 (23.9%)	
	II	73 (23.3%)		58 (25.7%)	
	III	18 (5.7%)			
	IV	4 (1.3%)			

**Mutation**	All negative			68 (30.1%)	
	ALK fusion			11 (4.9%)	
	EGFR mutation			127 (56.2%)	
	KRAS mutation			20 (8.8%)	
**Status**	Death : Alive	106:183 (36.7%:63.3%)	289	35:191 (15.5%:84.5%)	45:40 (52.9%:47.1%)
**Survival time**		52.08 months (0.20 - 190.40)	289	58.150 months (7.37 - 128.80)	68.00 months (0.00 - 221.00)

Supplementary Table 3. Significantly enriched gene ontology biological process terms of five survival-related network modules

Module		Description	p-value (FDR adjusted)
Black	✓	extracellular matrix organization	2.83E-34
	✓	extracellular structure organization	2.83E-34
	✓	extracellular matrix disassembly	2.13E-19
	✓	collagen metabolic process	1.18E-17
	✓	multicellular organismal macromolecule metabolic process	2.34E-17
Lightgreen	✓	interferon-gamma-mediated signaling pathway	4.66E-17
	✓	antigen processing and presentation of exogenous peptide antigen via MHC class I, TAP-dependent	3.05E-16
	✓	antigen processing and presentation of exogenous peptide antigen via MHC class I	3.82E-16
	✓	type I interferon signaling pathway	3.82E-16
	✓	cellular response to type I interferon	3.82E-16
Magenta	✓	mitochondrial translation	1.02E-02
	✓	mitochondrial translational initiation	1.02E-02
	✓	mitochondrial translational elongation	1.02E-02
	✓	mitochondrial translational termination	1.02E-02
Turquoise	✓	DNA strand elongation involved in DNA replication	7.59E-10

	✓	mitotic cell cycle phase transition	7.59E-10
	✓	DNA-dependent DNA replication	1.07E-09
	✓	cell cycle G1/S phase transition	1.63E-09
	✓	DNA strand elongation	4.21E-09
Red	✓	organic acid catabolic process	6.10E-03
	✓	carboxylic acid catabolic process	6.10E-03
	✓	small molecule catabolic process	9.60E-03
	✓	fatty acid metabolic process	1.03E-02
	✓	branched-chain amino acid catabolic process	1.03E-02

Supplementary References

[1] T. Barrett, D.B. Troup, S.E. Wilhite, P. Ledoux, C. Evangelista, I.F. Kim, M. Tomashevsky, K.A. Marshall, K.H. Phillippy, P.M. Sherman, NCBI GEO: archive for functional genomics data sets—10 years on, Nucleic acids research, 39 (2011) D1005-D1010.

[2] S.D. Der, J. Sykes, M. Pintilie, C.-Q. Zhu, D. Strumpf, N. Liu, I. Jurisica, F.A. Shepherd, M.-S. Tsao, Validation of a histology-independent prognostic gene signature for early-stage, non–small-cell lung cancer including stage IA patients, Journal of Thoracic Oncology, 9 (2014) 59-64.

[3] J. Hou, J. Aerts, B. Den Hamer, W. Van Ijcken, M. Den Bakker, P. Riegman, C. van der Leest, P. van der Spek, J.A. Foekens, H.C. Hoogsteden, Gene expression-based classification of non-small cell lung carcinomas and survival prediction, PloS one, 5 (2010) e10312.

[4] J. Botling, K. Edlund, M. Lohr, B. Hellwig, L. Holmberg, M. Lambe, A. Berglund, S. Ekman, M. Bergqvist, F. Pontén, Biomarker discovery in Non–Small cell lung cancer: Integrating gene expression profiling, meta-analysis, and tissue microarray validation, Clinical Cancer Research, 19 (2013) 194-204.

[5] R. Kuner, T. Muley, M. Meister, M. Ruschhaupt, A. Buness, E.C. Xu, P. Schnabel, A. Warth, A. Poustka, H. Sültmann, Global gene expression analysis reveals specific patterns of cell junctions in non-small cell lung cancer subtypes, Lung cancer, 63 (2009) 32-38.

[6] P. Micke, K. Edlund, L. Holmberg, H.G. Kultima, L. Mansouri, S. Ekman, M. Bergqvist, L. Scheibenflug, K. Lamberg, G. Myrdal, Gene copy number aberrations are associated with survival in histologic subgroups of non-small cell lung cancer, Journal of thoracic oncology, 6 (2011) 1833-1840.

[7] A.C. Borczuk, M. Sole, P. Lu, J. Chen, M.-L. Wilgus, R.A. Friedman, S.M. Albelda, C.A. Powell, Progression of Human Bronchioloalveolar Carcinoma to Invasive Adenocarcinoma Is Modeled in a Transgenic Mouse Model of K-ras–Induced Lung Cancer by Loss of the TGF-β Type II Receptor, Cancer research, 71 (2011) 6665-6675.

[8] L. Ding, G. Getz, D.A. Wheeler, E.R. Mardis, M.D. McLellan, K. Cibulskis, C. Sougnez, H. Greulich, D.M. Muzny, M.B. Morgan, Somatic mutations affect key pathways in lung adenocarcinoma, Nature, 455 (2008) 1069-1075.

[9] H. Okayama, T. Kohno, Y. Ishii, Y. Shimada, K. Shiraishi, R. Iwakawa, K. Furuta, K. Tsuta, T. Shibata, S. Yamamoto, Identification of genes upregulated in ALK-positive and EGFR/KRAS/ALK-negative lung adenocarcinomas, Cancer research, 72 (2012) 100-111.

[10] S. Rousseaux, A. Debernardi, B. Jacquiau, A.-L. Vitte, A. Vesin, H. Nagy-Mignotte, D. Moro-Sibilot, P.-Y. Brichon, S. Lantuejoul, P. Hainaut, Ectopic activation of germline and placental genes identifies aggressive metastasis-prone lung cancers, Science translational medicine, 5 (2013) 186ra166-186ra166.

[11] S. Davis, P.S. Meltzer, GEOquery: a bridge between the Gene Expression Omnibus (GEO) and BioConductor, Bioinformatics, 23 (2007) 1846-1847.

[12] L. Gautier, L. Cope, B.M. Bolstad, R.A. Irizarry, affy—analysis of Affymetrix GeneChip data at the probe level, Bioinformatics, 20 (2004) 307-315.

[13] R. Gentleman, V. Carey, W. Huber, F. Hahne, Genefilter: methods for filtering genes from high-throughput experiments, R package version, 1 (2015).

[14] W.E. Johnson, C. Li, A. Rabinovic, Adjusting batch effects in microarray expression data using empirical Bayes methods, Biostatistics, 8 (2007) 118-127.

[15] G. Yu, L.-G. Wang, Y. Han, Q.-Y. He, clusterProfiler: an R package for comparing biological themes among gene clusters, Omics: a journal of integrative biology, 16 (2012) 284-287.

[16] M.S. Cline, M. Smoot, E. Cerami, A. Kuchinsky, N. Landys, C. Workman, R. Christmas, I. Avila-Campilo, M. Creech, B. Gross, Integration of biological networks and gene expression data using Cytoscape, Nature protocols, 2 (2007) 2366-2382.

[17] L. Kang, W. Chen, N.A. Petrick, B.D. Gallas, Comparing two correlated C indices with right-censored survival outcome: a one-shot nonparametric approach, Stat Med, 34 (2015) 685-703.

[18] J. Katzman, U. Shaham, A. Cloninger, J. Bates, T. Jiang, Y. Kluger, Deep Survival: A Deep Cox Proportional Hazards Network, arXiv preprint arXiv:1606.00931, (2016).

[19] T. Tieleman, G. Hinton, Lecture 6.5-rmsprop: Divide the gradient by a running average of its recent magnitude, COURSERA: Neural Networks for Machine Learning, 4 (2012).

[20] F. Bastien, P. Lamblin, R. Pascanu, J. Bergstra, I. Goodfellow, A. Bergeron, N. Bouchard, D. Warde-Farley, Y. Bengio, Theano: new features and speed improvements, arXiv preprint arXiv:1211.5590, (2012).
